# Analysis of the whole transcriptome from gingivo-buccal squamous cell carcinoma reveals deregulated immune landscape and suggests targets for immunotherapy

**DOI:** 10.1371/journal.pone.0183606

**Published:** 2017-09-08

**Authors:** Richa Singh, Navonil De Sarkar, Sumanta Sarkar, Roshni Roy, Esita Chattopadhyay, Anindita Ray, Nidhan K. Biswas, Arindam Maitra, Bidyut Roy

**Affiliations:** 1 Human Genetics Unit, Indian Statistical Institute, Kolkata, India; 2 National Institute of Biomedical Genomics, Kalyani, India; Saint Louis University, UNITED STATES

## Abstract

**Background:**

Gingivo-buccal squamous cell carcinoma (GBSCC) is one of the most common oral cavity cancers in India with less than 50% patients surviving past 5 years. Here, we report a whole transcriptome profile on a batch of GBSCC tumours with diverse tobacco usage habits. The study provides an entire landscape of altered expression with an emphasis on searching for targets with therapeutic potential.

**Methods:**

Whole transcriptomes of 12 GBSCC tumours and adjacent normal tissues were sequenced and analysed to explore differential expression of genes. Expression changes were further compared with those in TCGA head and neck cohort (n = 263) data base and validated in an independent set of 10GBSCC samples.

**Results:**

Differentially expressed genes (n = 2176) were used to cluster the patients based on their tobacco habits, resulting in 3 subgroups. Immune response was observed to be significantly aberrant, along with cell adhesion and lipid metabolism processes. Different modes of immune evasion were seen across 12 tumours with up-regulation or consistent expression of *CD47*, unlike other immune evasion genes such as *PDL1*, *FUT4*, *CTLA4* and *BTLA* which were downregulated in a few samples. Variation in infiltrating immune cell signatures across tumours also indicates heterogeneity in immune evasion strategies. A few actionable genes such as *ITGA4*, *TGFB1* and *PTGS1/COX1* were over expressed in most samples.

**Conclusion:**

This study found expression deregulation of key immune evasion genes, such as *CD47* and *PDL1*, and reasserts their potential as effective immunotherapeutic targets for GBSCC, which requires further clinical studies. Present findings reiterate the idea of using transcriptome profiling to guide precision therapeutic strategies.

## Introduction

Gingivo-buccal squamous cell carcinoma (GBSCC) is one of the most prevalent types of oral cancers in India and most common etiologic factors are tobacco and alcohol use [1]. But ~10% of all GBSCC incidences can be partially explained by human papilloma virus (HPV) infection[2]. Previous mutation exploration studies on HNSCC revealed several high impact mutations resulting in molecular heterogeneity [3–4]and highlighted variants associated with longer disease free survival[5]. Deeper molecular understanding on other tiers of molecular heterogeneity (e.g. mRNA expression) could be a key for effective therapeutic options to enhance overall disease free survival. Transcriptome profiling could shed light on many avenues of such clinically relevant molecular marks, including expression deregulation, disrupted molecular/biological pathways and detection of fusion transcripts. Earlier transcriptome studies on oral cancer have put forward deregulation signatures of *HOX* families and adherens junction genes as well as the infiltrating immune cells in head and neck malignant tissues as compared to premalignant and normal tissues [6]. Our study has elucidated the immunoregulatory gene expression landscape in a specific site of oral cancer, i.e., GBSCC primary tumours, as compared to their adjacent normal tissue and computationally estimated relative composition of various immune cell classes in those tissues. A detailed portrayal of expression variation of immune evasion genes could illuminate target genes for potential immunotherapy which has not been yet addressed. Further, to broaden our knowledge on expression regulation, we expanded our analysis to look at correlated miRNA expression of deregulated genes. Despite a small sample set (n = 12), the resulting characterization of transcriptome profiles from the Indian GBSCC case series will be very useful to guide newer avenues of precision therapies for this globally infrequent, but most prevalent, oriental oral cancer type.

## Materials and methods

### Study design and sample collection

This study was approved by the Review Committee for Protection of Research Risks to Humans, Indian Statistical Institute, Kolkata. Unrelated patients diagnosed with GBSCC in oral cavity were selected during 2009 to 2012 from the Guru Nanak Institute of Dental Sciences and Research, a tertiary dental college and hospital at Kolkata, India. Informed consent was obtained from all participants (n = 12) for use of tissue samples in this study. All patients were personally interviewed to get information on age, sex, occupation, alcohol consumption, type of tobacco habits, frequency and duration of their daily tobacco usage and place of work. Sample collection was performed in accordance with the relevant guidelines of Institute’s ethical committee. Tumour samples, confirmed histopathologically as GBSCC, and adjacent control tissue were included in the study (Table A in S2 File). Choice of adjacent control tissue from same patient was intentional to minimize inter-individual differences in tobacco exposure and the affected tissue site.

### HPV infection

*VirusSeq* algorithm[7], which uses subtraction method to detect viruses and integration sites, was applied to identify patients with or without infection of any virus such as HBV, HCV, HIV including HPV16 and HPV18. The *VirusSeq* uses a catalogue of 26512 viruses from GIB-V (genome information broker for viruses) database and check for their presence and integration sites in paired-end RNAseq data given to it. It uses a subtraction based method similar to *PATHSeq*.

### Sample processing and library preparation

Total RNA was isolated from 12 pairs of tumours and adjacent normal tissues followed by column-based *DNase* treatment procedure (Qiagen Inc., USA). RNA preparation, with integrity number (RIN) ≥ 7, was selected for study and ribosomal *RNA* was removed followed by enzymatic fragmentation. Double-stranded cDNA was prepared from RNA and bar-coded adapters were ligated as per library preparation protocol (Illumina Inc., USA). Average fragment size and quantification of libraries were performed in bioanalyzer and qPCR (Kapa quantification kit), respectively. Bridge amplification was performed and paired-end sequencing was done in HiSeq 2000 (Illumina Inc.) using TruSeq Paired-end chemistry to generate (100+100) bp length reads.

### Data processing and differential gene expression analysis

Initial quality check of the sequence data was performed in FASTQC. Reads were aligned using splice-aware aligner, *STAR*, in paired-end mode with Gencode v19 transcriptome assembly and the *hg19* genome reference. Number of reads uniquely mapped (mapping quality ≥20) to defined genic coordinates were then counted with *HTSeq* (union mode). To check for library count bias, disease vs. normal, *logCPM* (i.e. counts per million) plots were performed before and after consistent expression and low expression filter (less than 50 reads per gene per million mapped reads). Lowly/inconsistently expressed genes were removed from further analysis.

Differential expression analysis was performed using *EdgeR* which used total library size of each sample for read count normalization across all genes [8]. The *EdgeR* package used empirical *Bayesian* model to compute biological covariance and *GLM* fitting approach to sort out the differentially expressed genes with likelihood ratio test in the context of paired samples. Deregulated expression of genes was considered as significant when p-value < 0.05 (FDR corrected) and at least 1.5-fold change in expression were observed.

### Validation

Expression of a subset of 37 nuclear and 4 mitochondrial DNA encoded genes was validated in another set of 10 GBSCC samples by SYBR green qPCR assay. The reason to choose expression of these genes is that we are, also, looking at the role of mitochondrial function related genes in oral cancer, in a separate ongoing study. So, we choose to validate expression of these 41 genes, which were, also, deregulated in transcriptome study.

### Pathway enrichment analysis

Differentially expressed genes were investigated in *GSEA* to identify altered pathways adopting *KEGG*, *Reactome* and *Biocarta* gene sets. Enrichment analysis was done separately for each set of genes and the p-value threshold was kept at 0.05 (FDR corrected) (Table B in S2 File). Significantly altered biological processes were identified using gene ontology annotations with a p-value cut off 0.05 (FDR corrected).

### Immune cell composition analysis

We applied “robust enumeration of immune cell subsets analysis” using *Cibersort* [9] on gene length normalized total expression data (FPKM). *Cibersort’s* standard immune cell reference data was used as the training set.

### Cell cycle proliferation score (CCP)

We have extrapolated CCP score estimation method [10] using log transformed FPKM expression values of 31 cell cycle activity associated signature genes. Cumulative Z score estimation followed by scale normalization was performed to obtain percent proliferation score for each sample.

### Analysis of correlation between expression of miRNA and target mRNA

We have previously reported the miRNA expression profile of 10 pairs of samples which are, also, a sub-set of 12 pairs of samples used in this transcriptome study [11]. Two-tailed paired t-test (FDR at p-value 0.05) was performed on the miRNA expression profile from these 10 samples to generate a list of miRNAs whose expression was significantly deregulated. Target mRNAs of the deregulated miRNAs were then explored from the list of 2176 deregulated mRNAs in present study.

### Chimeric genes identification

Potentially targetable or actionable fusion genes were, also, identified by the *Fusion Catcher* [12] pipeline. These findings were further cross-validated using the *ChimeraScan* [13] pipeline. All observed fusions are supported by at least 10 reads spanning the fusion site. Using *BreakDancer* [14] pipeline, translocation breakpoints were identified from exome sequencing data of same sample set (unpublished data).

## Results

### Differential expression profile

Of 53million paired-end reads per sample, on an average 49million paired-end reads were successfully aligned to the human reference genome version *hg19* (Table C in S2 File). Transcripts from all samples were found to be free of HPV16, HPV18 or any other viruses by the *VirusSeq* pipeline [7]. Using normalized mRNA expression data of 8845 consistently expressed genes, we performed multidimensional scaling (MDS) based sample clustering (each gene’s expression was considered as independent vector, distance metric: Euclidian). Evidently, diseased tissue mRNA expression formed a distinct cluster from normal tissue along the vertical axis (Figure A in S1 File). The majority of normal tissues formed a tighter cluster as compared to tumor tissues, which were seen to have a wider spread both along the vertical and horizontal axis. Such observations are indicative of gross inter-tumour expression heterogeneity. Even though 2N seems to be closer to 25D in the sample cluster, the former is shown to be plotted at a distance from its tumor tissue(2D) counterpart. Similarly, 17N and 17D were plotted at distance. Comparison of differentially expressed genes was done using pair-wise model (i.e. tumor of a sample was compared against its adjacent normal). So, even if the expression profile of a tumor seems to be closer to the expression profile of a normal tissue from a different individual, the analysis of differential gene expression (comparing the adjacent normal from the same individual) is unaffected.

**Fig 1:**
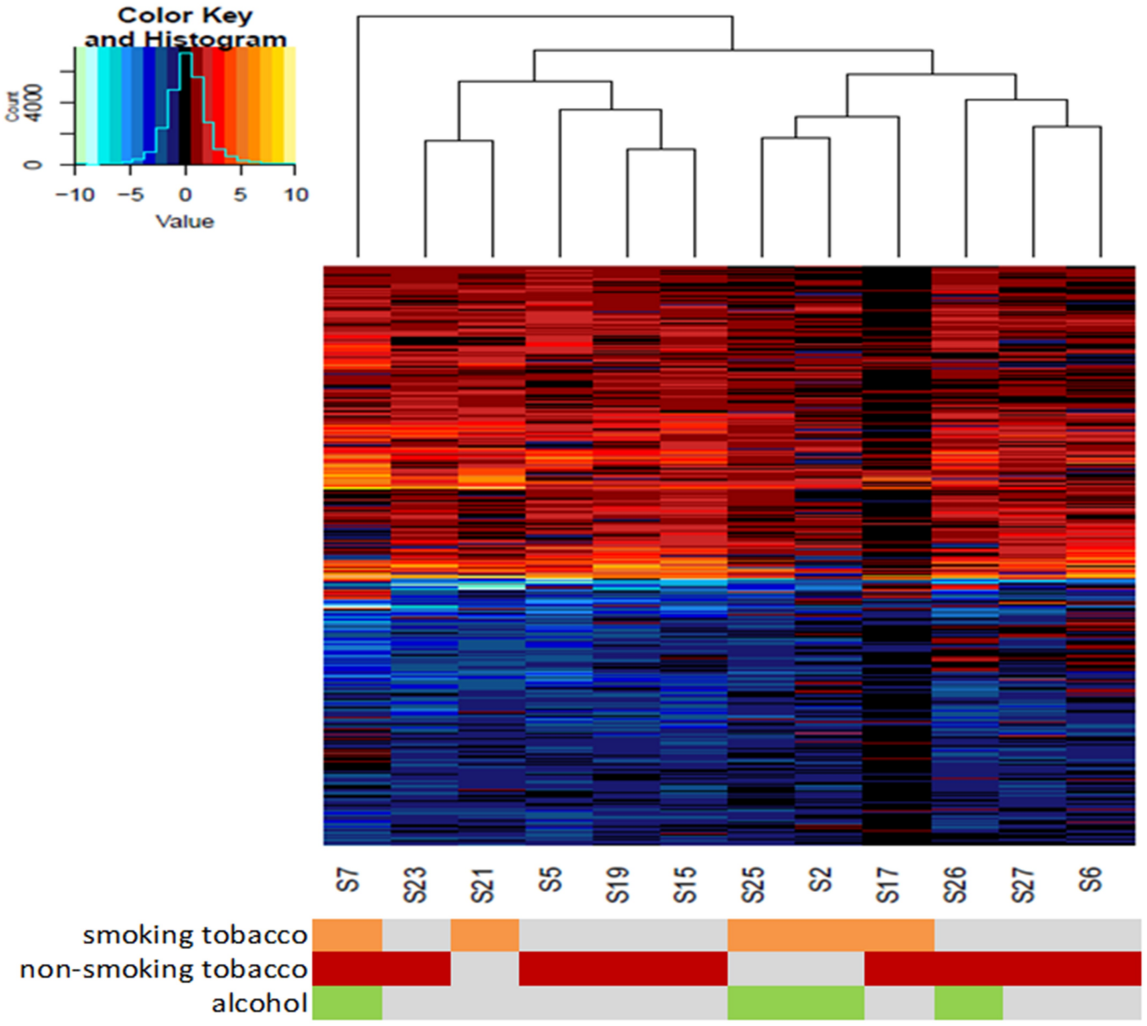
Hierarchical clustering of GBSCC tumours by 2176 deregulated gene expressions. Hierarchical clusters were constructed based on log2 transformed expression values of 1002 upregulated (represented by colours of negative values in heatmap) and 1174 downregulated genes (represented by colours of positive values in heatmap). Across all 12 tumours there is a gross similarity in deregulation pattern, with some exceptions. As a result, 3 distinct sample clusters were noticed. The coloured panel below, represent subject's smoking (orange) and/or chewing tobacco (red) and/or alcohol abuse (green)status.

We observed expression of 2176 genes to be significantly deregulated in cancers compared to its respective paired normal tissue (with FDR correction of p-value 0.05 and at least 1.5 folds change in expression) (Table D in S2 File, Figure B in S1 File). Among these 2176 genes, expression of 1002 genes was up regulated and that of 1174 genes was downregulated. An unsupervised clustering (Fig 1), using the expression of all deregulated genes (n = 2176), divided the 12 cancer samples into two major and one minor subgroups. The minor subgroup comprised only one tumour (S7) and this patient had tobacco smoking, chewing as well as alcohol consumption habit. One of the two major subgroups comprised tumours from five patients (S23, S21, S5, S19 and S15) who mostly had a history of non-smoking/chewing tobacco habit (Table A in S2 File). The third subgroup comprised 6 tumours and these patients (S25, S2, S17, S26, S27 and S6) had either tobacco smoking or chewing or mixed habit. Furthermore, S17 sample shows lower level of deregulated expression compared to other samples which might be due to effect of unknown factors in this patient. Expression of 41 (37 nuclear and 4 mitochondrial DNA encoded) genes, amongst these 2176 genes, were further validated by qPCR/RT-PCR (SYBR green method) in an independent set of 10 samples (Table E in S2 File) and, except 3 nuclear DNA encoded genes, the trend of expression deregulation was similar to our whole transcriptome based observation.

### Pathway analysis

Both cell adhesion and immune surveillance associated pathways were consistently enriched across all four gene sets in *GSEA* (Table B in S2 File) (*Biocarta*, *KEGG*, *Gene Ontology*:*Biological processes and Reactome*). Other pathways/processes such as lipid metabolism and PPARA (known to regulate lipid metabolism) were also enriched in two gene sets (*Reactome*; FDR p-value 2.98E^-22^ and *Biocarta*; FDR p-value 3.33E^-06^).

### Immunoregulatory signatures and cell cycle progression marks

**Fig 2:**
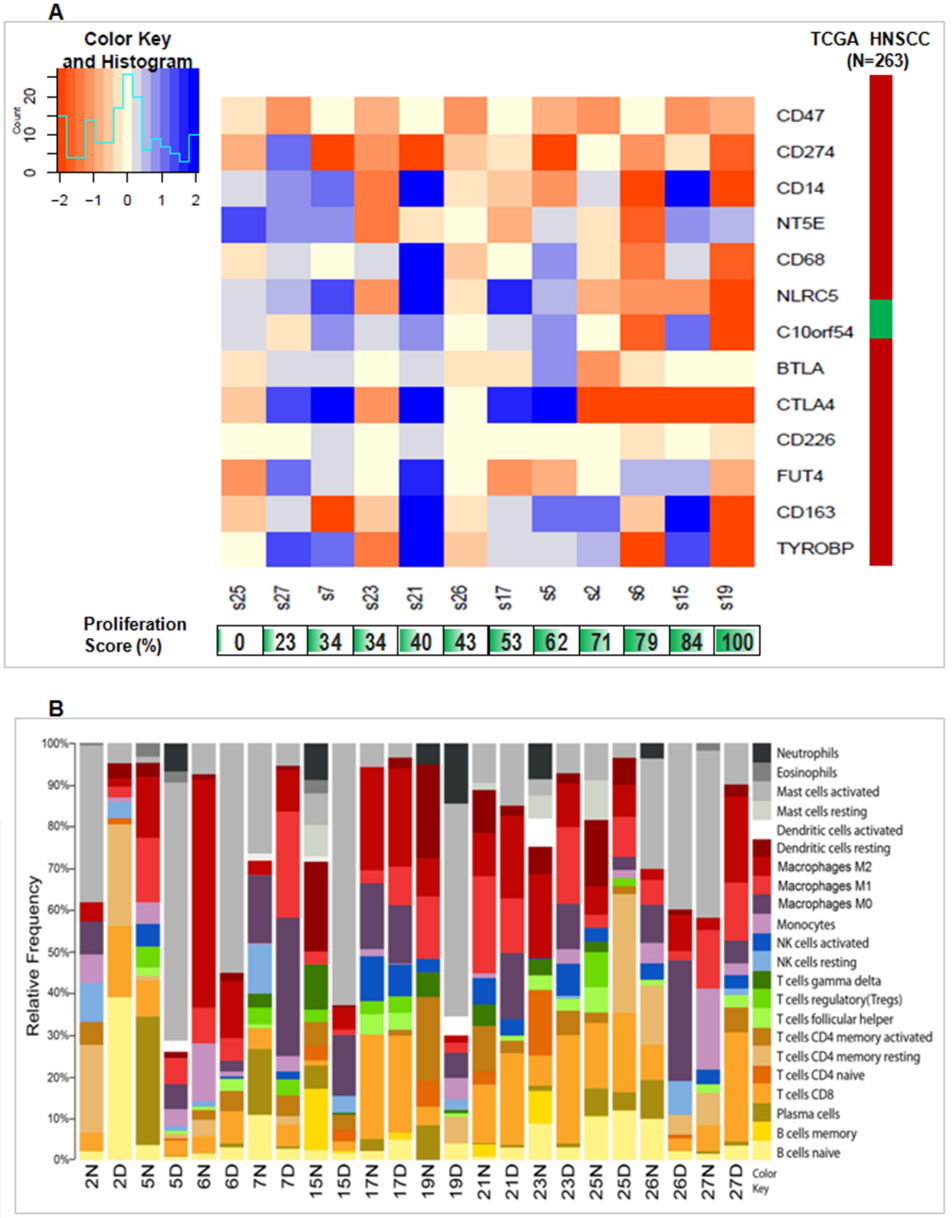
Immune response alterations in tumor compared to normal tissues. (A) Heatmap shows diverse expression levels (log fold change of FPKM values) of immune evasion genes. Orange values in color bar shows up regulation while values in blue show down regulation. The panel below shows proliferation scores per samples with green color intensity indicating higher proliferation score and numbers indicates % CCP score. The right-side panel indicates fold change values (FPKM) in TCGA HNSCC cohort (n = 263) for each gene. In case of TCGA HNCC tissues, Green color denotes downregulation while red color shows upregulation in the right-side panel. (B) The barplot shows how relative composition of immune cells is altered across 12 pairs of tumor compared to its control tissues. Plot was derived from the *CIBERSORT* [9] estimated data output from FPKM normalized expression data. Every color stands for a type of immune cell and height of each colored bar represents relative frequency of an immune cell type. In the plot, 2N and 2D indicate normal and tumour tissues of tumour-normal paired S2sample, respectively. Similar nomenclature was used for tumour and normal tissues of other samples.

As immunotherapy is a rising actionable and patient-specific treatment, we investigated any potential for various immunotherapeutic modalities within the tumour subsets. It is already appreciated that HNSCC oncogenesis and progression is strongly associated with profound immune defects, as mutational accumulation allows cancer cells to evade immune-surveillance. GBSCC is no exception, as immune surveillance loss could result from the elevated expression levels of *CD47* across most of the studied tumours (Fig 2A). Furthermore, *CD274* (i.e. *PD-L1*) was significantly up-regulated in most of the tumours but downregulated in only two tumours. Expression of another 11 immuno-regulatory genes showed remarkable heterogeneity across tumours. Deregulation of *CD47* and *CD274*is in concordance with an earlier report of 263 TCGA tumours but remaining 11 immuno-regulatory genes showed heterogeneous deregulation. A majority of genes within immune-evasion pathways were upregulated in tumours S6, S19 and S23 (Fig 2A), whereas majority of the same genes were seen to be downregulated in S27 and S21. Expression of *CTLA4* and *NLRC5* was consistently upregulated in samples with a high proliferation score (Fig 2A; calculated on the basis of expression of 31 proliferation markers [10]. Contrarily, S27 and S21 tumours had very low and moderate proliferation scores. Interestingly, *CD47* had a significant positive correlation (FDR at 0.05 p-value) with expression of many of these proliferation markers, excluding *BUB1B*, *MCM10*, *PBK*, *RRM2*, *RAD51* and *RAD54L* (Table F in S2 File). On an average, all of the genetic markers for cell cycle progression were upregulated in our cancer series, similar to the report in the TCGA HNSCC tumours (Table F in S2 File). Beyond these cell cycle proliferation markers, elevated expression of genes associated with anti-apoptosis or cell cycle progression (*PCNA*, *CDC25B* and *BIRC3)*, angiogenesis (*ELF4*, *PLCG2*, *SHC1*, *POSTN* and *DOK2)* and downregulation of anti-angiogenic signalling (*ANGPTL1*), together relays another tier of accelerated cell growth signatures in these tumours (Table D in S2 File).

Expression alteration of immune evasion genes led to the curiosity of exploring variation in immune cell composition in tumours. Using length normalized transcriptome data (FPKM), we have computationally predicted relative frequency of several immune cell populations in the tumour tissues (Fig 2B). We used *CIBERSORT’s [9]* relative cell population prediction mode and noticed heterogeneous frequency of immune cell populations. To our surprise, we found a higher relative frequency of activated mast cell population in tumors (5D, 6D, 15D, 19D, 21D, 23D and 26D) of 7 tumor-normal paired samples (S5, S6, S15, S19, S21, S23 and S26) while tumors (2D, 7D, 17D, 25D and 27D) of the remaining 5 tumor-normal paired samples (S2, S7, S17, S25 and S27) showed lower relative frequencies compared to respective normal tissue. Success of several immunotherapeutic modalities were often associated with the frequencies of infiltrating CD8^+^-T-cells or M0, M1 and M2 macrophages. Interestingly, we observed distinctive dichotomies in our set of samples and detected 5 tumours (2D, 6D, 21D, 23D and 27D) of 5 tumor-normal paired samples (S2, S6, S21, S23 and S27) with relatively higher CD8^+^-T-cell population frequency than their respective normal tissues (2N, 6N, 21N, 23N and 27N). For other samples, the frequency of CD8^+^-T-cells were either lower in tumours (5D, 19D and 26D) of tumour-normal paired samples (S5, S19 and S26) or similar in tumours of (7D, 15D, 17D and 25D) of tumor-normal paired samples (S7, S15, S17 and S25) compared to its normal counterpart. Again, we predicted 4 of the tumors (7D, 17D, 23D and 25D) of tumor-normal paired samples (S7, S17, S23 and S25) to have higher M1 macrophage frequency and5 tumors (7D, 15D, 21D, 26D and 27D) of tumor-normal paired samples (S7, S15, S21, S26 and S27) to have higher M2 macrophage frequency compared to its paired normal tissue.

### Altered expression of tissue architecture related genes

In the pathway enrichment study, ECM maintenance was seen to be one of the most enriched biological processes. Abnormal ECM dynamics can lead to deregulated cell proliferation, invasion, avoidance of cell death, and loss of cell differentiation, making this pathway a promising potential target for therapy. In this study, we observed consistent elevated expression of key components of this pathway, such as *matrix-metallo-proteinases* and *ADAMs* across all 12 tumors, indicating substantial involvement of these proteins in gingivo-buccal cancer (Table G in S2 File). Barring a few genes (*ITGA9*, *ITGA7*, *COL14A1*, *LAMB2* and *LAMA2)*, expression of many components of cell adhesion, focal adhesion, proteoglycans, integrins, collagens and laminins were upregulated. Guanine nucleotide exchange factors (GEFs), such as VAV proteins for the Rho family of *GTPases*, activate a pathway that leads to actin cytoskeletal rearrangements and transcriptional alterations. Consistent up-regulation of *VAV1* and *VAV2* observed across all tumours, was also replicated in a previous report [15]. Interestingly *TIMP3*, an inhibitor of matrix metalloproteinases [16], was also found to be downregulated in these tumours. Upregulated expression of several matrix metalloproteinase genes as well as the altered expression of several integrin genes, is a common molecular feature of GBSCC that could be an additional target for treatment since protein of few such upregulated genes, were further reported to be expressed in HNSCC tissues mentioned in Human Protein Atlas database[17].

### Lipid metabolism

Lipid and glucose metabolic pathway homeostasis was disrupted in these tumours, emphasizing that, as seen in liver cancer, these pathways may be critical in oral cancer as well [18–19]. It has been reported that activation of *PPARGC1A* may enhance fatty acid oxidation and insulin sensitivity in muscle cells[20]. Likewise, in these cancer samples, the downregulation of *PPARGC1A* may also contribute to downstream of insulin resistance and reduced mitochondrial fatty acid oxidation. Previous research indicates that low *ACACB* expression (involved in the *AMPK* pathway) is possibly due to low expression of*PPARGC1A* and *PPARA* (Table D in S2 File). Changes in expression of *CD36* and *PPARA* indicate that fatty acid oxidation and cholesterol metabolism may also be suppressed. Therefore, the observed signals for fatty acid oxidation, involving *PPARGC1A*, *ACACB*, *CD36* and *PPARA*, were observed to be repressed in GBSCC. Further, suppression of fatty acid oxidation, influenced by downregulation of *SCP2*, *ACOX1*, *ACOX3* and the possible suppression of cholesterol metabolism is supported by an observed low expression of *CYP27A1*. Expression of *EPHX2*, which is responsible for the catabolism of arachidonic acid [21], was downregulated in all GBSCC samples. Phospholipases, such as *PLA2G16*, *PLA2G12A* and *PLA2G4A* were not highly expressed in the tumour tissues. Reduced *PPARGC1A* expression may indirectly promote gluconeogenesis and reduce blood glucose uptake. Glycogen synthesis might also be impacted due to activation of *IRS1* and *PIK3CD*. AMP-activated protein kinase (*AMPK*) is a master regulator of cellular energy homeostasis and is activated in response to a high ADP:ATP ratio. Our result suggests that the low expression of *EEF2K* and *CAB39L* are additional primary factors which may lead to inactivation of *AMPK* pathway in GBSCC, as reported earlier [22].

### miRNA and expression of target genes

**Fig 3:**
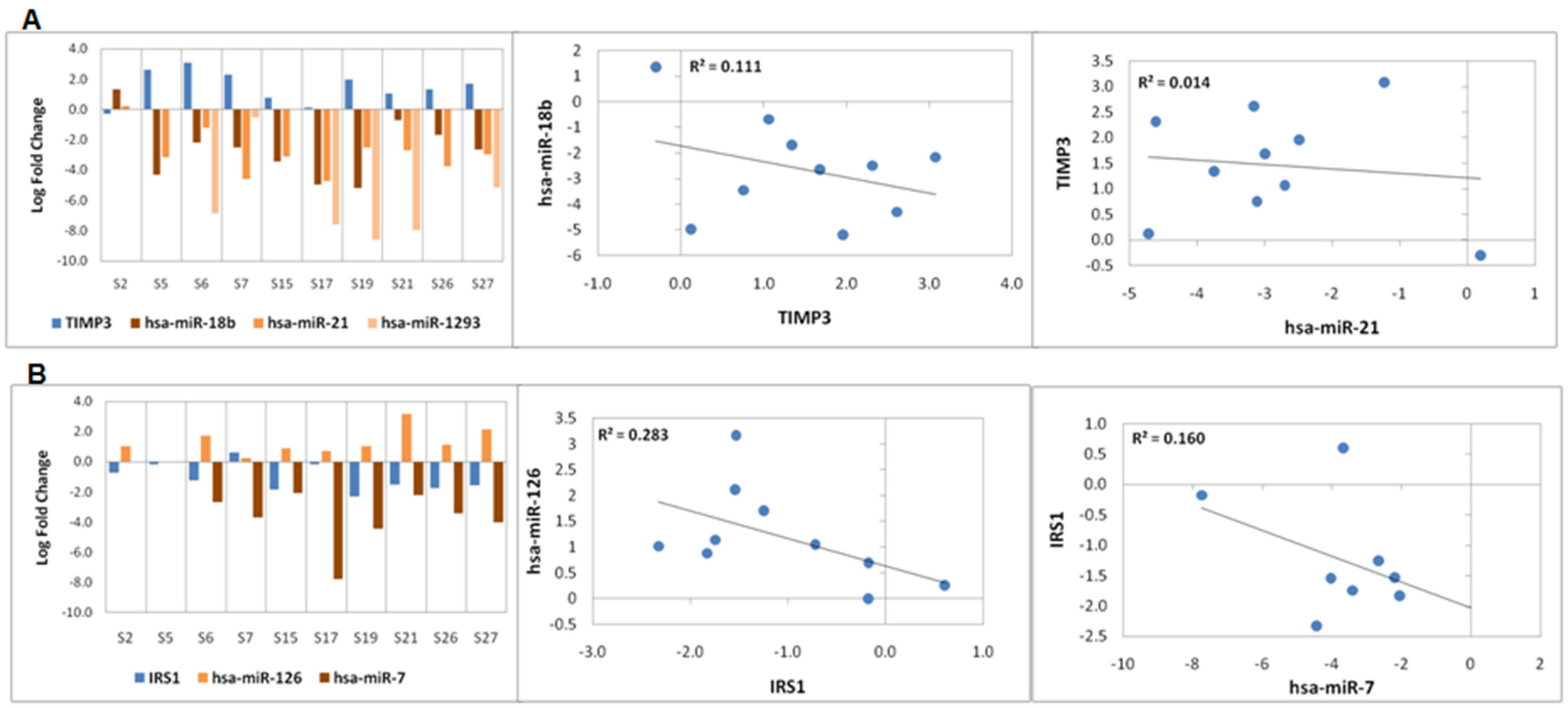
Expression deregulation of miRNA and target mRNAs. (A) Bar plots show log_2_fold change in expression of miRNAs (hsa-miR-18b, hsa-miR-1293and hsa-miR-21) and their target, TIMP3. Scatter plots show negative correlation of TIMP3 expression with hsa-miR-18b and hsa-miR-21expression. Negative correlation was not observed between TIMP3 and hsa-miR-1293. Negative values of log_2_fold change indicate upregulated expression whereas positive log_2_fold change values indicate downregulation. (B) Bar plot showslog_2_fold change of expression of miRNAs (hsa-miR-126 and hsa-miR-7) and their target, IRS1. Scatter plot shows negative correlation of IRS1 expression with hsa-miR-126 and hsa-miR-7expression. Negative values of log_2_fold change indicate upregulated expression whereas positive fold change values indicate downregulation.

In a previous study [11], we profiled miRNA expression deregulation in 18 oral cancer samples which includes 10 samples used in this study. Only 77 genes in this RNASeq dataset seem to be under strong regulation of the 24 miRNAs (Table H in S2 File) deregulated in these 10 samples. Few proteoglycans and gap junction genes, such as *TIMP3* (targeted by *hsa-miR-1293*, *hsa-miR-18b-5p* and *hsa-miR-21-5p*), *TUBA1B* (targeted by *hsa-miR-140-3p*), *PLAU* (targeted by *hsa-miR-23b-3p*), were seen to be deregulated in an opposite direction compared to their targeting miRNAs (Fig 3 and Figure C in S1 File). We did not find any significantly deregulated miRNAs that target immunoregulatory genes in these tumours. Other prominent miRNA regulated pathways in GBSCC include fatty acid metabolism (target genes: *GRB10*, *LRP6*, *ACADVL* and *ACAT1*) and sugar metabolism (target genes: *IRS1* and *IRS2*), suggesting that there may be additional unexplored biological roles of these miRNAs in carcinogenesis. Negative correlation was observed between expression of *TIMP3* and *IRS1* with their respective targeting miRNAs (Fig 3). This suggests a possible regulation of gene expression by miRNAs in tumours.

### Transcribed fusion gene profiles

**Fig 4:**
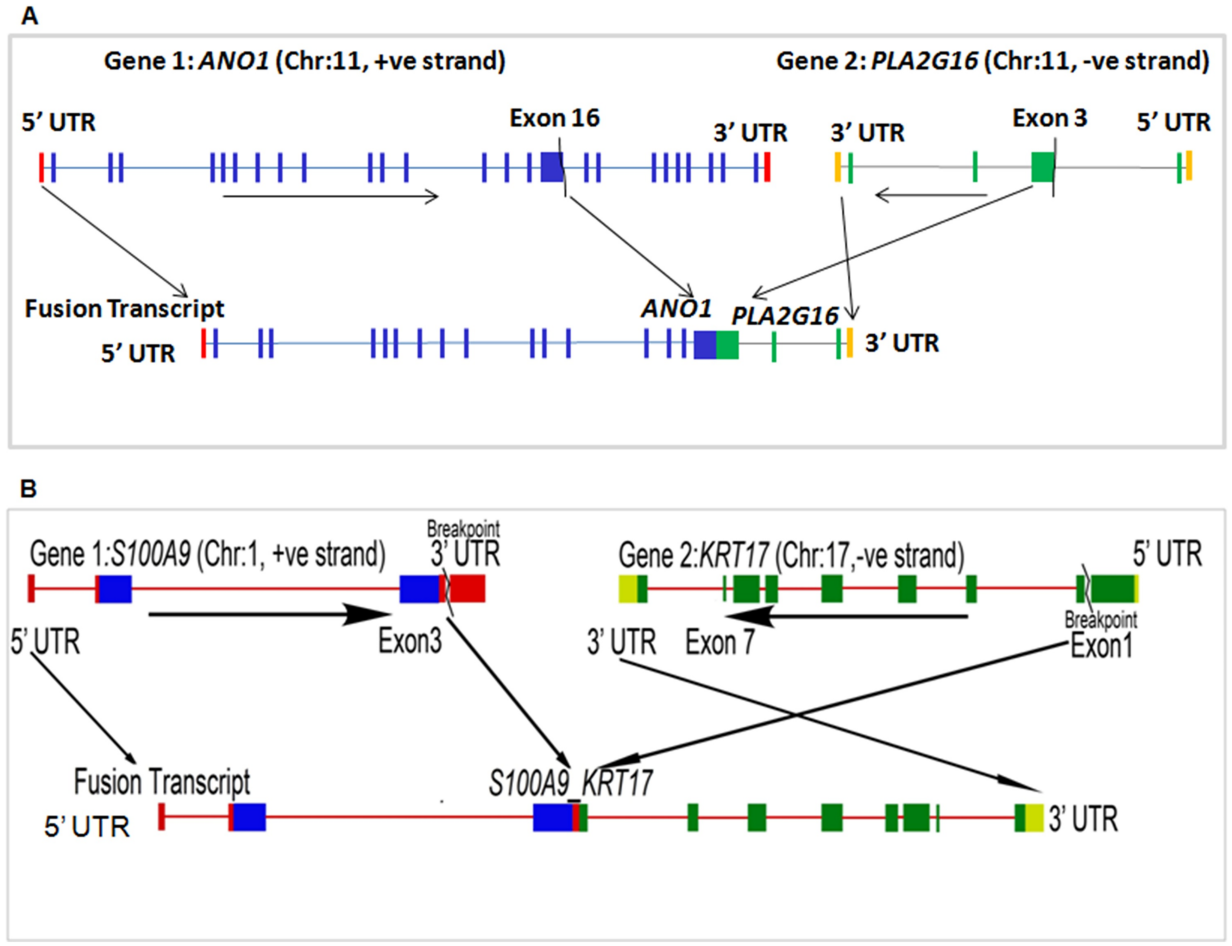
Schematic view of fusion events. (A) ANO1-PLA2G16 fusion gene which retains exon 16 along with upstream exons of ANO1 and exon 3 along with downstream exons of PLA2G16 in tumour tissue (i.e. 23D) of the tumour-normal paired S23 sample. (B) S100A9-KRT17 fusion gene deduced from coding regions up to exon 3 of S100A9 and exon 1 to all other downstream exons of KRT17 in tumour tissue (i.e. 2D) of the tumour-normal paired S2 sample.

Using the *Fusion Catcher* [12] pipeline for reliable fusion event detection, only three somatic events could be identified. Considered parameters were based on sequence similarity and known variable regions (Table I in S2 File). Genes involved in cell adhesion (*KRT17*) and lipid metabolism (*PLA2G16*) harboured fusion breakpoints in few samples. In tumour tissue of S23 sample, i.e.23D, *PLA2G16* (in arachidonic acid metabolism pathway) was found to be fused with *ANO1*, known to promote an aggressive phenotype in breast cancer. Fusion events were further checked using the *Chimerascan* tool [13] (Table J in S2 File). Fused transcripts include exon 16 of *ANO1* (including both cytoplasmic and trans-membrane domains) and a portion of exon 3 of *PLA2G16* (including a cytoplasmic domain with an active enzyme site) (Fig 4A). We observed two additional fusion events between *S1000A9* and *KRT17*, which has previously been reported in the TCGA database (Fig 4B and Table I in S2 File). Activation of *S100A9* is also associated with breast cancer metastatic progression, whereas *KRT17* is a potent oncogene. Interestingly in the TCGA Pan-Cancer data, these two genes were highly expressed and substantially upregulated in head and neck cancers [23]. In this study, expression of *KRT17* was upregulated, especially in two tumour tissues (2D and 25D) of paired tumour-normal samples S2 and S25, respectively, detected to have this fusion. The fusion is predicted to retain the coding regions up to exon 3 of *S100A9*, and part of exon 1 to all other exons of *KRT17*. Elevated expression of *KRT17* in these two samples could partially be explained by this fusion, as it might be using active promoter of *S100A9*.

Attempts were made to validate the fusion events by PCR and RT-PCR in the DNA and cDNA, respectively, from the respective samples but the fused PCR products could not be detected suggesting that fusion did not happen in 100% cells of the tumours. So, the tumours contained both fused and wild type genes and transcripts, since wild type genes (*ANO1 and PLA2G16)* were amplified by PCR (data not shown) from DNA of the sample but no PCR product was observed for the fused gene using proper primers. Similarly, no RT-PCR fusion product was observed from cDNA made from the tumour RNA. However, using NGS technique (i.e. exome sequencing data) on the same samples, translocation events were identified in same tumour tissues. Translocation breakpoints in tumour DNA were detected near to fusion points, i.e. *ANO1-PLA2G16* and *S100A9-KRT17* fusion transcripts, in same samples (Table K in S2 File, Table L in S2 File).

## Discussion

It is now evident that the oncogenesis of oral cancer involves the loss of function of many tumour suppressors as well as the accumulation of profound immune defects, leading to downstream homeostatic imbalances and eventual immune evasion [24–25]. As the role of the immune system in cancer becomes better understood, several potential immunotherapeutic modalities for OSCC have come to fruition. Such immunotherapies must confer both a durable response and increased survival for patients. A recent finding has highlighted changes in the immune response and cell composition during the progression from dysplasia to cancer [6]. Building from this finding, the present study sheds light on the importance of transcripts as another tier of molecular heterogeneity. It is now appreciated that the molecular contexts, specifically the tumour-specific immunoregulatory signatures, are essential to assess appropriate immunotherapeutic modalities. As such, a better understanding of molecular contexts could provide more precise stratification of patient groups in clinical trials. Our investigation elucidated a spectrum of molecular heterogeneity that might be relevant to certain modes of immunotherapies.

We explored the molecular marks of tumour-specific immune evasion signature by means of altered mRNA expression in samples of oral cancer. One well-studied immune evasion mechanism involves the interaction of tumour surface-localized*CD47*withmacrophage or dendritic surface-localized *SIRPα* (*SIRPA*), conveying a “don't-eat-me” signal [26–27]. Furthermore, *CD47* mRNA expression levels are correlated with a decreased probability of survival for multiple types of cancer. Blockade of *CD47* signalling via targeted monoclonal antibodies enables macrophage to phagocytose vulnerable tumour cells that were previously protected from immune surveillance.

Programmed death 1 *(PD-1)* is an immune inhibitory receptor expressed by *PDCD1* on several immune cells, particularly cytotoxic T-cells, which can result in an immune evasion phenotype. *PD-1* interacts with the ligand, programmed death ligand 1 (*CD274*), expressed on tumour as well as other immune cells, to inhibit T-cell activation and cytokine production, eventually leading to tumour immune evasion [28–29]. Our results indicate that these immune responses may be controlled by a variation in expression of different immune evasion phenotype linked genes. The gene *CD47* was found to be upregulated or highly expressed in all samples, but for the remaining 12 genes, mRNA expression pattern was heterogeneous (Fig 2A). Among the 12 genes, expression level of *CD274 (i*.*e*. *PDL1)* was upregulated in 10 of the 12 studied tumours (Fig 2A); suggesting these subsets of tumours may be susceptible for checkpoint blockade therapy. Efficacy of certain immunotherapeutic strategies, such as inhibition of immune checkpoints i.e. *PD-L1* blockade, often depend on the concentration of tumour infiltrating lymphocytes (TILs) and CD8^+^-T-cells in the tumour microenvironment. A higher CD8^+^-T:CD4^+^-T ratio, in addition to PD-L1 blockade, may warrant an effective therapeutic outcome [30].

Several other molecular and clinical parameters that may indicate a better response to a *PD-L1* blockade strategy include total mutation burden, histology (squamous epithelial differentiation status), frequencies of TILs, *PD-L2* expression, ratio of T-reg and CD8^+^-T-cell frequencies, lymphoid aggregates, and degree of tumour necrosis. Here, we demonstrated an application of RNASeq data to determine tumour immune signatures that could potentially facilitate precision medicine practices such as immunotherapy. We adopted the *CIBERSORT* algorithm to compute the relative frequency of various immune cells from the total transcriptome data. Tumour tissues from S21 and S23 samples had high CD8^+^-T:CD4^+^-T (TIL) ratios, indicating that these tumours may likely respond to the immune checkpoint blockade therapy as mentioned above. Although data from oral cancer-specific immunotherapy trials is not yet available, ongoing clinical trials conducted with lung cancer patients have found several responders with lack of *PD-L1* expression, as well as responders with low mutation burdens and no history of smoking abuse. Furthermore, there was also observed poor response associated with some of the above-mentioned checkpoint blockade markers, including the TIL frequency, *PD-L2* expression, *CD8*^+^*-T*:*CD4*^+^*-T* ratio, lymphoid aggregates, and necrosis status [30]. Although GBSCC or oral cancer clinical trial data is not yet published, a drive for novel and predictive molecular markers is vital to highlight the importance of multi-tier molecular data generation.

Thirty-one expressed cell cycle proliferation (CCP) markers were used to calculate a proliferation score (Table F in S2 File). Tumours with greater proliferation or faster cell cycle turnover were likely to have a more aggressive form of GBSCC and a poorer prognosis [31]. As such, tumours with the highest proliferation scores are expected to have the most prominent marks of immune evasion. If we consider the high expression of the observed 13 immunoregulatory genes as a measure of immunoevasive signal strength, then we can find that expression of most of these genes was upregulated in tumours with high proliferation scores (samples S6 and S19) (Fig 2A). *CTLA4* and *NLRC5* expression was consistently upregulated in all samples with a high proliferation score (>70%). Correlation between expression (i.e. FPKM values) of the 13 immunoevasion phenotype regulatory genes and 31 proliferation markers was studied but only *CD47* showed positive correlation with most of the proliferation genes (Table F in S2 File) suggesting its stronger influence on cell proliferation than other immune evasion genes.

Disruption of the tissue architecture maintenance, via altered cell adhesion, as well as *AMPK* and *PPAR* pathways, were observed in these primary GBSCCs tissues. Consistent deregulation of several genes in these pathways lead to observed GSEA enrichment of glucose and lipid metabolism pathways in our data. Many of the genes, whose expression was significantly deregulated, are either current or potential clinically actionable targets (Table M in S2 File). Due to lack of resources, we could not study protein expression of deregulated genes in our samples, but compared the RNA expression of the study with immuno-histochemistry data in HNSCC tissues (Human Protein Atlas) [17] and RPPA data in oral cancer tissues from TCGA [32] (Table M in S2 File, Table N in S2 File). Up- and down-regulation of several genes in our samples corroborate with their protein expression, including actionable gene such as *LCK*, in oral cancer tissues from TCGA database (Table N in S2 File). Proteins of other actionable genes (Table M in S2 File) along with few immune evasion genes such as *CD47*, *NT5E*, *C10ORF54*, *CD226* and *FUT4* were observed to be expressed in HNSCC tissues in Human Protein Atlas database (data not shown). Genes involved in cell adhesion, proteoglycan, *PPAR* and *AMPK* signalling pathways or genes like *PTGS1* are known targets of available inhibitors or drugs [33]. Expression of genes included in these pathways, such as *ITGA4*, *CD274*, *PIK3CD* and *TGFB1* was upregulated in almost all samples and could be considered as future targetable genes in GBSCC. Further in-vitro and in-vivo preclinical functional studies are needed for a better understanding of such possibilities. Besides, factors, like loss of expression of *CBR1*, involved in several oncology response metabolism, or an increase in expression of *ABCC4*, associated with drug resistance [34], should also be considered during prioritizing various treatment options for a patient [35].

Evidence of upregulated miRNAs and inversely expressed target transcripts has also highlighted potentials for even more therapeutic target nodes in oral cancer. *TIMP3*, known to inhibit metalloproteases [16], was downregulated in the cancer tissue samples and is known to be a target of miRNAs. These miRNAs (*hsa-miR-1293*, *hsa-miR-18b-5p*and *hsa-miR-21-5p*) were expectedly seen to be upregulated in the set of studied tumour tissues. Although the window of possibilities with miRNA guided therapies are narrow, such consistent observations highlight the possibilities of oral cancer treatment with a few specific anti-miRNAs against *hsa-miR-1293*, *hsa-miR-18b-5p* and *hsa-miR-21-5p* [36].

As chimeric antigen receptors on T-cell (CAR-T) based approaches are making progress in medicine [37–38], the search for specific and targetable neo-antigens gained importance in order to facilitate effective therapeutic options. Tumour-specific gene-gene fusion derived chimeric proteins might turn out to be effective antigen for specific antitumor therapy. Three samples had fusion events in this study but two of them had fusion between *S100A9* and *KRT17*. This fusion appeared to increase the expression of *KRT17*, which might in turn facilitate progression to a more aggressive disease phenotype. Thus, such a fusion can have a prognosis prediction marker value in disease and may be considered as a candidate neo-antigen for a new generation therapy.

However, it is apparent that within all clinically actionable genes, there remains a large variation of expression across different tumour tissues. Even after targeting a specific gene with a specific agent, different patients may respond differentially. Considering the substantial heterogeneity within the tier of mRNA expression, it will be increasingly important to combine specific therapeutic modalities along with functional immunotherapy. Such combined approaches may initiate more sustainable and effective treatment options for GBSCC.

In conclusion, our study showed landscape of deregulated expression of genes in GBSCC which essentially includes genes related to immunity, cell adhesion and lipid metabolism. It also proposes potential therapeutic targets depending on the altered expression profiles. Further, an understanding of expression of these genes and their plausible regulation by miRNAs might be important for its clinical application. Evidences put forward by this study fortify the application of immuno-transcriptome in precision therapy.

## Supporting information

S1 File Figure A: MDS Plot with 12 pairs of tumour and normal samples. Multidimensional scaling plot of 12 pairs of tumour-normal samples using expression of 8845 genes. The sample clustering showed a distinction between disease and its paired normal tissues. The plot shows 12 pairs of samples where 2N and 2D indicated normal and tumour tissues of sample S2, respectively. Similar nomenclature was, also, used for tumour and normal tissues of other paired samples. Figure B: Smear plot represents average log fold change in expression of all genes. Average log fold change of all transcripts (n = 57,818) with which differential expression analysis was performed. Red dot above and below two black lines (i.e. central zones) indicates the fold changes of significantly deregulated expressed genes (n = 2176). Figure C: Expression change of miRNAs and their target mRNAs across 10 cancer samples. The plot shows log_2_fold change in expression of the miRNAs and its respective target mRNAs from cell-adhesion, glucose metabolism and lipid metabolism processes across 10 sample pairs which were common in current transcriptome and previous miRNA studies. Values with negative log_2_ fold change signify upregulation while those with positive values signify downregulation.
(DOCX)

S2 File Table A: Demography of the patients. Table B: Enrichment of biological pathways using BIOCARTA, KEGG, Reactome and Gene Ontology (GO:BP) in GSEA portal. Table C: Read counts of raw reads and aligned reads across all samples. Table D: List of genes with deregulated expression having log₂ fold change (CPM values) in each sample and average fold change, red font gene mentioned in text. Table E: Fold change in expression of 37 nuclear and 4 mitochondrial DNA encoded genes in a different set of 10 oral cancer tissue by RT-PCR SYBR green method. Table F: Correlation between FPKM values of CD47 and 31 proliferation marker genes with FDR corrected p-values. Table G: Differentially expressed genes in cell adhesion and related pathways with average fold change. Table H: Expression deregulation of 77 mRNAs regulated by 24 unique miRNAs (some miRNAs target more than one mRNAs). Table I: Fusion events were examined across all samples by *Fusion Catcher pipe line*. Table J: Fusion events were examined across all samples by *Chimerascan pipeline*. Table K: Comparison of fusion breakpoint in RNA data (by *Fusion Catcher*) and translocation breakpoint in DNA data (by *Breakdancer)* from same tumour tissue. Table L: Comparison of fusion breakpoint in RNA data (by *Chimerascan*) and translocation breakpoint in DNA data (by *Breakdancer*) from same tumour tissue. Table M: List of deregulated target genes with known drugs and protein expression in HNSCC samples from Human Protein Atlas. Table N: Comparison of RNA expression in our samples with protein expression in oral cancer samples in TCGA cohort and HNSCC tissues in Human Protein Atlas.
(XLSX)
