# Supplementary material for: Analysis of the whole transcriptome from gingivo-buccal squamous cell carcinoma reveals deregulated immune landscape and suggests targets for immunotherapy
Source: PLoS One. 2017 Sep 8;12(9):e0183606. doi: 10.1371/journal.pone.0183606 (PMC5590820; doi:10.1371/journal.pone.0183606)
Supplement: S1 File — Figure A: MDS Plot with 12 pairs of tumour and normal samples. Multidimensional scaling plot of 12 pairs of tumour-normal samples using expression of 8845 genes. The sample clustering showed a distinction between disease and its paired normal tissues. The plot shows 12 pairs of samples where 2N and 2D indicated normal and tumour tissues of sample S2, respectively. Similar nomenclature was, also, used for tumour and normal tissues of other paired samples. Figure B: Smear plot represents average log fold change in expression of all genes. Average log fold change of all transcripts (n = 57,818) with which differential expression analysis was performed. Red dot above and below two black lines (i.e. central zones) indicates the fold changes of significantly deregulated expressed genes (n = 2176). Figure C: Expression change of miRNAs and their target mRNAs across 10 cancer samples. The plot shows log2fold change in expression of the miRNAs and its respective target mRNAs from cell-adhesion, glucose metabolism and lipid metabolism processes across 10 sample pairs which were common in current transcriptome and previous miRNA studies. Values with negative log2 fold change signify upregulation while those with positive values signify downregulation. (DOCX) [file pone.0183606.s001.docx]

**Supplementary Figures**

**Figure A: MDS Plot with 12 pairs of tumor and normal samples**

**Legend:** Multidimensional scaling plot of 12 pairs of tumor-normal samples using expression of 8845 genes. The sample clustering showed a distinction between disease and its paired normal tissues. The plot shows 12 pairs of samples where 2N and 2D signifies normal and tumor tissues of sample S2 respectively.

**Figure B: Smear plot represents average log fold change in expression of all genes.**


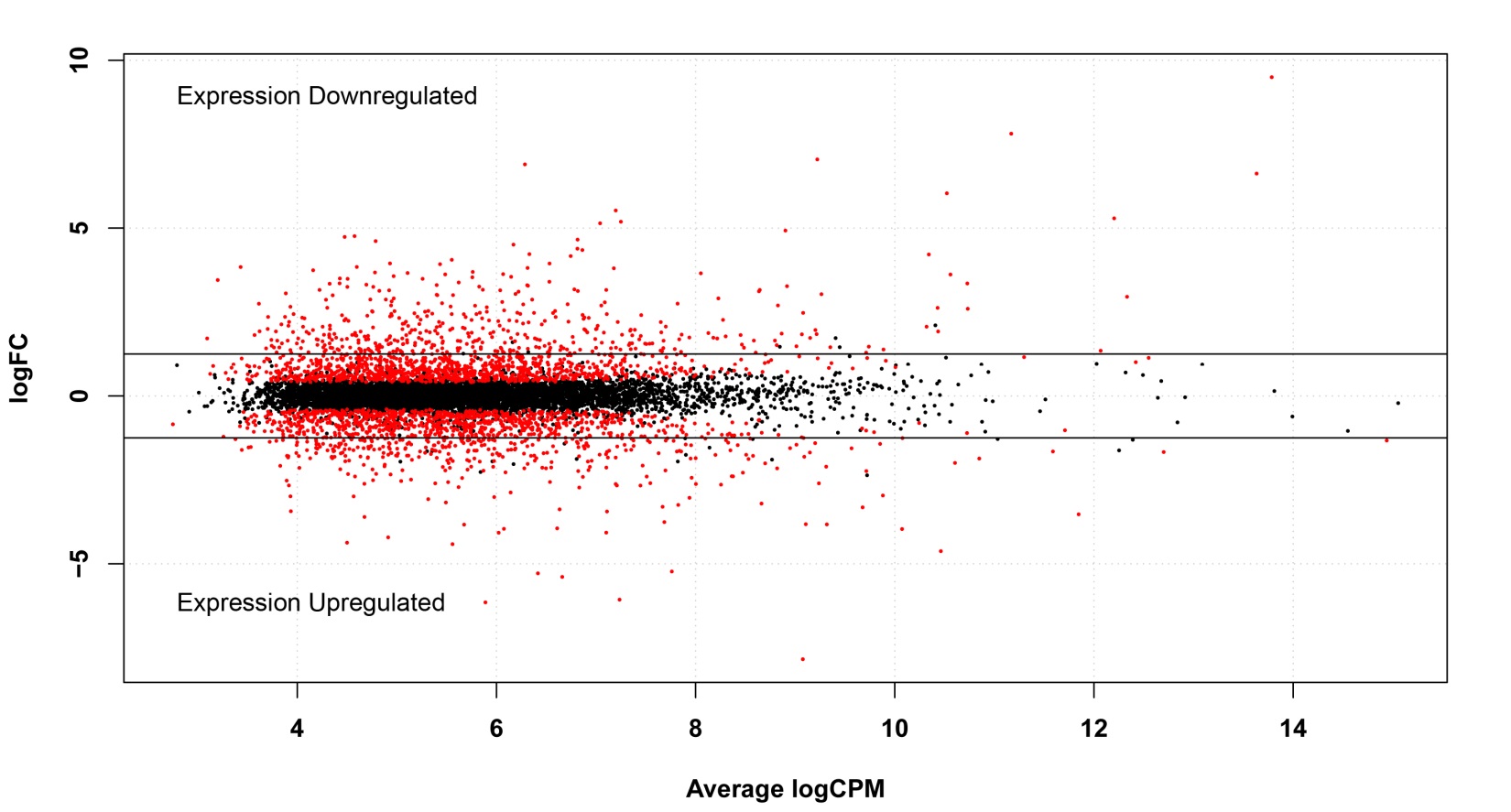


**Legend:** Average log fold change of all transcripts (n=57,818) with which differential expression analysis was performed. Red dot above and below two black lines (i.e. central zones) indicates the fold changes of significantly deregulated expressed genes (n=2176).

**Figure C: Expression change of miRNAs and their target mRNAs across 10 common sample pairs.**

**
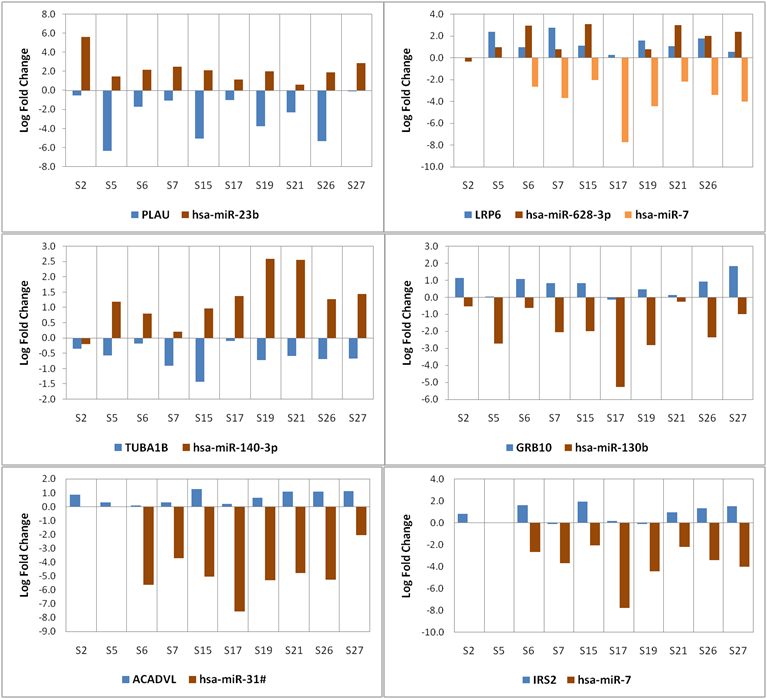
**

**Legend:**  The plot shows log fold change in expression of the miRNAs with its respective target mRNAs from cell-adhesion, glucose metabolism and lipid metabolism processes across 10 sample pairs which were common in present and previous miRNA study. Values with negative log fold change signify upregulation while those with positive values signify downregulation.
